# RPLC Determination of Tinidazole and Diloxanide Furoate in Tablets

**DOI:** 10.4103/0250-474X.45415

**Published:** 2008

**Authors:** P. N. S Pai, G. K. Rao, B. Srinivas, S. Puranik

**Affiliations:** Department of Quality Assurance, Al-Ameen College of Pharmacy, Bangalore-560027, India

**Keywords:** Tinidazole, Diloxanide furoate, HPLC, UV detection

## Abstract

A simple reverse phase liquid chromatographic method has been developed and subsequently validated for simultaneous determination of tinidazole and diloxanide furoate. The separation was carried out using a mobile phase consisting of acetonitrile, methanol and 0.2 M potassium dihydrogen phosphate (pH 5) in the ratio 2:3:2.The column used was SS Wakosil-II C-18 with a flow rate of 1 ml/min and UV detection at 282 nm. The described method was linear over a concentration range of 10-70 μg/ml and 10-90 μg/ml for the assay of diloxanide furoate and tinidazole, respectively. The mean recovery was found to be 100-101% for tinidazole and 97-103% for diloxanide furoate when determined at three different levels.

Tinidazole and diloxanide furoate as components of a multi-ingredient formulation is very useful in therapy of diarrhoea due to amoebiasis and associated with mixed infections by bacteria. As found from the literature, diloxanide furoate has been reported to be estimated in combination with other drugs by HPLC^1^–^3^ methods. Tinidazole has been reported to be estimated by HPLC^4^–^6^ methods. Tinidazole and diloxanide furoate have been simultaneously determined by spectrometric methods^7^–^10^. The aim of the present work is to describe a liquid chromatographic procedure for the separation and simultaneous quantification of tinidazole and diloxanide furoate in its formulation.

For the proposed method all the chemicals of analytical reagent grade, solvents of HPLC grade and distilled water (Millipore) were used. The LC system consisted of LC-10AT pump (Shimadzu), SS Wakosil-II C-18, 250×4.6 mm, 5 μm column, Rheodyne injector equipped with a 100 μl sample loop and UV detector (Shimadzu SPD-10A VP) set at 282 nm. The output signal was monitored and integrated using CZ-RA software (Shimadzu).

The standard solution of diloxanide furoate 500 μg/ml and tinidazole 500 μg/ml were prepared separately by dilution of tinidazole and diloxanide furoate respectively in mobile phase of acetonitrile, methanol and 0.2M potassium dihydrogen phosphate pH 5.0 in the ratio 2:3:2.

Analysis of marketed sample Amicline Plus (Laboratories Griffon Pvt. Ltd.) of three different batches was carried out. Twenty tablets, each containing 375 mg diloxanide furoate and 300 mg tinidazole were weighed. The tablets were crushed together in a mortar to a fine powder and an amount equivalent to 375 mg diloxanide furoate and 300 mg of tinidazole was transferred into a 100 ml dried volumetric flask. A few drops of acetonitrile were added to dissolve the active solids and then volume made up with the mobile phase. The solution was degassed through membrane filter. The standard solution and sample solutions were injected separately into the stabilized liquid chromatographic system. The retention time for tinidazole and diloxanide furoate at a flow rate of 1ml/min were recorded as 3.4 and 5.2 min respectively (fig. 1). From the respective peak areas obtained in standard and sample chromatogram. The amount of contents was calculated. The results of the analysis are tabulated in Table 1.

**Fig 1:**
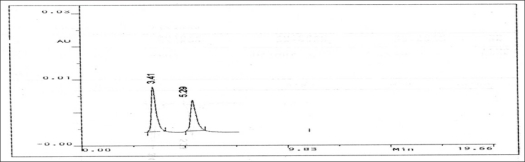
Chromatogram of marketed formulation The Retention time for tinidazole and diloxanide furoate at 3.41 min and 5.29 min

**TABLE 1:** ASSAY FOR TINIDAZOLE AND DILOXANIDE FUROATE

Batch	Content of diloxanide furoate (mg/tab)	Amount of diloxanide furoate found*(mg/tab)	Content of tinidazole (mg/tab)	Amount of tinidazole found*(mg/tab)
Bt-1	375	371.5	300	309.5
Bt-2	375	370.5	300	307.5
Bt-3	375	374.4	300	306.0

* Calculated from six replicate analysis of three separate batches of marketed formulation

Accuracy of the method was checked by recovery studies, where in sample was spiked with known quantity of standard drug of tinidazole and diloxanide furoate at 3 different levels of 80, 100 and 120% each. The percentage recovery ranged from 100-101% for tinidazole and 97-103% for diloxanide furoate. The precision of the method was studied by analysis of the mixture and expressed as percentage relative standard deviation, which was found to be 0.01% for diloxanide furoate and 0.02% for tinidazole.

The linearity of the method was established by analysis of standard solution. The calibration curve was drawn by plotting the peak area versus concentration. The linearity range was found to be 10-70 μg/ml for diloxanide furoate and 10-90 μg/ml for tinidazole. The specificity of the method was established by injecting placebo. No interference of the placebo was observed with the principal peaks. Ruggedness of the method was determined by carrying out the experiment on different instruments, by different chemists and on different days. The results showed that the method was rugged as percentage recovery was found to be in the range of 95.1-100.1%. Robustness of the method was determined by making slight changes in the chromatographic conditions. Buffer pH modification by ±5% did not have any significant effect. The effect of organic strength on retention time was studied by small change in percentage polarity of the mobile phase system and it was found that even slight percentage change, upto 10% in ratio of mobile phase did not alter the position of the peaks.

The system suitability tests were carried out as per USP XXIV requirements. System suitability tests were carried out on freshly prepared standard stock solution of tinidazole and diloxanide furoate and the parameters obtained with 100 μl injection volume. The number of theoretical plates for tinidazole and diloxanide furoate was calculated as 71790 and 74444, respectively. The symmetry factor for tinidazole and diloxanide furoate peaks was 1.05 and 0.91, respectively. The resolution between the two peaks was 1.92. The obtained results confirmed that the method is highly suitable for its intended purpose of separation of tinidazole and diloxanide furoate and its simultaneous determination in formulation.
